# Supplementary material for: Sphingosine-1-Phosphate (S1P) Lyase Inhibition Aggravates Atherosclerosis and Induces Plaque Rupture in ApoE−/− Mice
Source: Int J Mol Sci. 2022 Aug 24;23(17):9606. doi: 10.3390/ijms23179606 (PMC9455951; doi:10.3390/ijms23179606)
Supplement: Supplementary file 1 [file ijms-23-09606-s001.zip › ijms-1826928-supplementary.pdf]

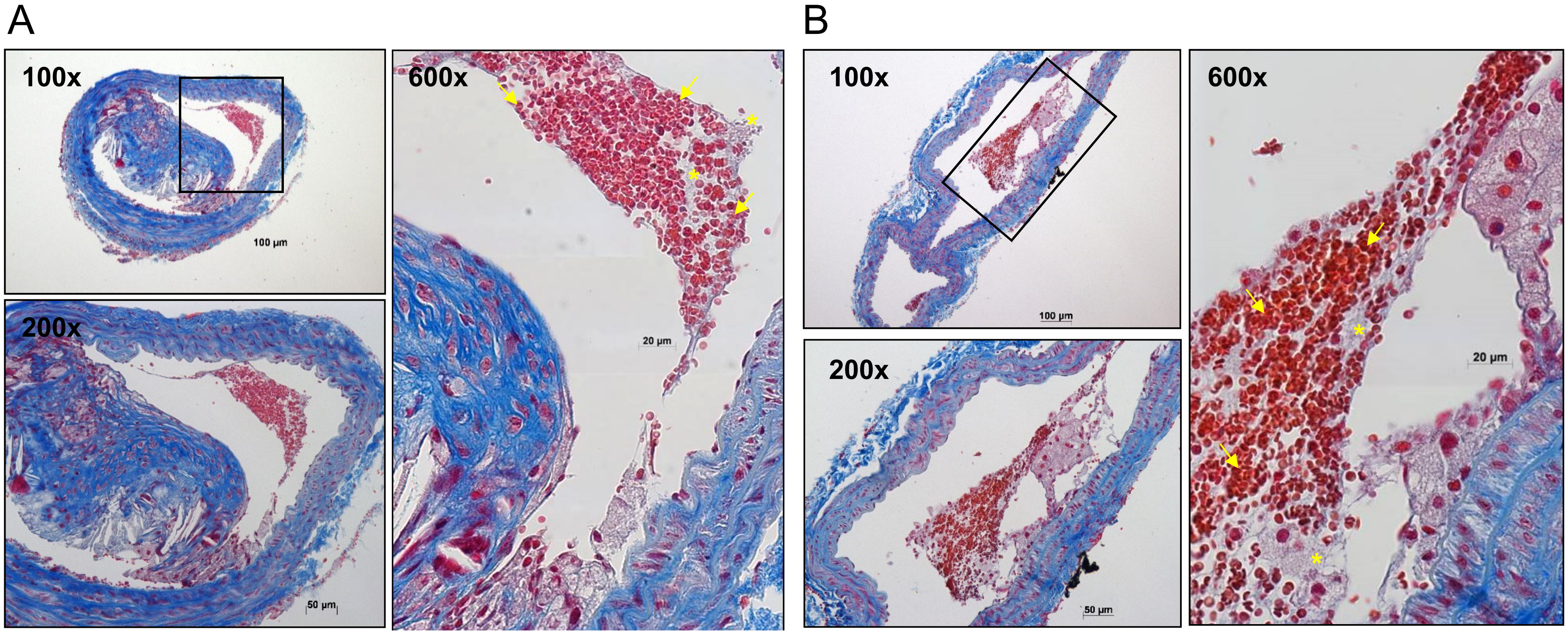

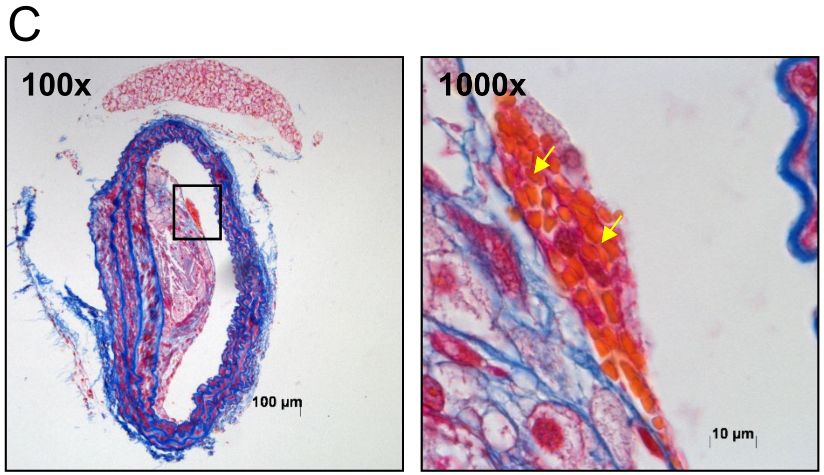

Supplemental Figure S1. Representative lesions from DOP-treated ApoE$^{-/-}$mice demonstrate multiple features of thrombosis. Representative BCA lesions from ApoE$^{-/-}$ mice on a Western type+DOP diet for 12 weeks stained with Carstairs and featuring different types of plaque rupture and thrombosis.(A-C). A and B, Superficial rupture of the foam cell layer with connection to a thrombus consisting of erythrocytes (→) and platelets (∗). Magnification as indicated. C, Atherosclerotic lesion of a ApoE$^{-/-}$ control mouse fed a Western type diet for 12 weeks showing a very small accumulation of erythrocytes (→) as part of a tiny mixed thrombus on top of the lesion. Magnification as indicated.

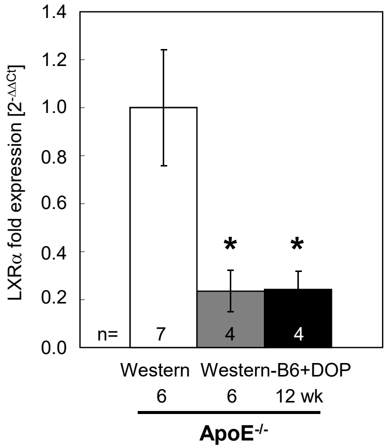

Supplemental Figure S2. Real-time PCR analysis of LXR αin macrophages of peritoneal macrophages isolated from ApoE$^{-/-}$mice on Western versus Western-B6+DOP diet for 6 and 12 weeks, respectively. *$p<0.05$.

Cytokines 1 week	$\Delta Ct_{control}$	ΔCt_{DOP}	p value
MCP-1	16.1	15.8	n.s.
CCR-2	6.4	6.5	n.s.
TNF-α	9.6	8.8	n.s.
IFN-γ	12.5	13.2	n.s.
IL-1	8.9	7.6	n.s.
IL-4	13.8	13.6	n.s.
IL-6	12.8	14.1	n.s.
IL-8	10.2	11.2	n.s.
IL-10	11.9	11.6	n.s.
IL-12	11.8	10.9	n.s.
TGF-β	0.9	0.9	n.s.
GM-CSF	12.5	11.9	n.s.
RANTES	12.0	11.1	n.s.
CCR-1	4.5	5.3	n.s.
Arg-1	2.0	1.8	n.s.
iNOS	18.0	17.7	n.s.

Cytokines 6 weeks	$\Delta Ct_{control}$	ΔCt_{DOP}	p value
TNF-α	9.2	9.9	n.s.
Arg1	3.6	3.9	n.s.
CCR5	16.7	16.4	n.s.
CCR7	11.7	11.6	n.s.
CXCR1	16.3	16.8	n.s.

Supplemental table S1. Real-time PCR analysis of inflammation-associated markers in thioglycollate-elicited peritoneal macrophages of *ApoE*$^{-/-}$ mice on a Western type diet compared to macrophages from mice on Western-B6+DOP for 1 and 6 weeks, respectively.
